# Comprehensive Review on Alzheimer’s Disease: Causes and Treatment

**DOI:** 10.3390/molecules25245789

**Published:** 2020-12-08

**Authors:** Zeinab Breijyeh, Rafik Karaman

**Affiliations:** Pharmaceutical Sciences Department, Faculty of Pharmacy, Al-Quds University, Jerusalem 20002, Palestine; z88breijyeh@gmail.com

**Keywords:** Alzheimer’s disease, neurodegeneration, β-amyloid peptide, tau protein, risk factors, disease-modifying therapy, chaperons, heat shock proteins

## Abstract

Alzheimer’s disease (AD) is a disorder that causes degeneration of the cells in the brain and it is the main cause of dementia, which is characterized by a decline in thinking and independence in personal daily activities. AD is considered a multifactorial disease: two main hypotheses were proposed as a cause for AD, cholinergic and amyloid hypotheses. Additionally, several risk factors such as increasing age, genetic factors, head injuries, vascular diseases, infections, and environmental factors play a role in the disease. Currently, there are only two classes of approved drugs to treat AD, including inhibitors to cholinesterase enzyme and antagonists to *N*-methyl d-aspartate (NMDA), which are effective only in treating the symptoms of AD, but do not cure or prevent the disease. Nowadays, the research is focusing on understanding AD pathology by targeting several mechanisms, such as abnormal tau protein metabolism, β-amyloid, inflammatory response, and cholinergic and free radical damage, aiming to develop successful treatments that are capable of stopping or modifying the course of AD. This review discusses currently available drugs and future theories for the development of new therapies for AD, such as disease-modifying therapeutics (DMT), chaperones, and natural compounds.

## 1. Introduction

**Figure 1:**
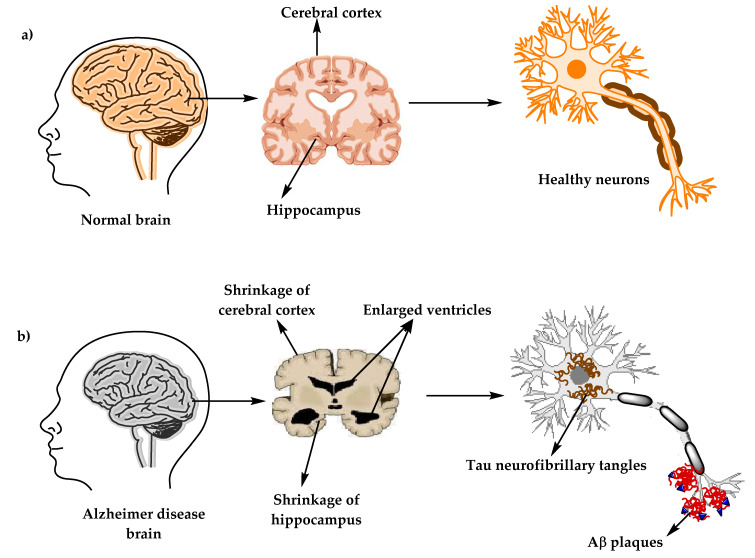
The physiological structure of the brain and neurons in (**a**) healthy brain and (**b**) Alzheimer’s disease (AD) brain.

Alzheimer’s disease (AD) (named after the German psychiatric Alois Alzheimer) is the most common type of dementia and can be defined as a slowly progressive neurodegenerative disease characterized by neuritic plaques and neurofibrillary tangles (Figure 1) as a result of amyloid-beta peptide’s (Aβ) accumulation in the most affected area of the brain, the medial temporal lobe and neocortical structures [1]. Alois Alzheimer noticed a presence of amyloid plaques and a massive loss of neurons while examining the brain of his first patient that suffered from memory loss and change of personality before dying and described the condition as a serious disease of the cerebral cortex. Emil Kraepelin named this medical condition Alzheimer’s disease for the first time in his 8th edition psychiatry handbook [2,3]. Progressive loss of cognitive functions can be caused by cerebral disorder like Alzheimer’s disease (AD) or other factors such as intoxications, infections, abnormality in the pulmonary and circulatory systems, which causes a reduction in the oxygen supply to the brain, nutritional deficiency, vitamin B12 deficiency, tumors, and others [4,5].

At present, there are around 50 million AD patients worldwide and this number is projected to double every 5 years and will increase to reach 152 million by 2050. AD burden affects individuals, their families, and the economy, with estimated global costs of US$1 trillion annually. At present, there is no cure for Alzheimer’s disease, although there are available treatments that just improve the symptoms [6,7]. The purpose of this review is to give a brief description about AD diagnosis, pathology, causes, and current treatments, and to highlight the recent development of compounds that could prevent or treat AD by targeting several pathogenic mechanisms, such as Aβ and tau aggregation, and misfolding, inflammation, oxidative damage, and others.

## 2. Alzheimer’s Disease Diagnostic Criteria

A patient suspected to have AD should undergo several tests, including neurological examination, magnetic resonance imaging (MRI) for neurons, laboratory examinations such as vitamin B12, and other tests besides the medical and family history of the patients [8]. Vitamin (vit.) B12 deficiency has been long known for its association with neurologic problems and increasing risks of AD, according to some studies. A special marker of vit. B12 deficiency is elevated homocysteine levels, which can cause brain damage by oxidative stress, increasing calcium influx and apoptosis. Diagnoses of vit. B12 deficiency can be done by measuring serum vit. B12 level alongside complete blood count and serum homocysteine levels tests [9,10].

In 1984, The National Institute of Neurological and Communicative Disorders and Stroke (NINCDS) and the Alzheimer’s Disease and Related Disorders Association (ADRDA) formed a work group (NINCDS-ADRDA) to establish a clinical diagnostic’s criteria for Alzheimer’s disease. This criteria includes: (1) probable Alzheimer’s disease, which can be diagnosed by dementia that is confirmed by neuropsychological tests, progressive memory loss, impaired daily-life activity, and other symptoms like aphasia (impairment of a language), apraxia (a motor skills disorder), and agnosia (a loss of perception). All of these symptoms can start from age 40–90, with the absence of any systemic or brain diseases, (2) possible Alzheimer’s disease can be applied in the absence of neurologic, psychiatric disorders, and the presence of another illness like systemic or brain disorder, but they are not the primary cause of dementia, and (3) definite Alzheimer’s disease, that is confirmed by histopathologic confirmation obtained from a biopsy or autopsy [11,12].

In 2011, The National Institute on Aging—Alzheimer’s Association made several changes and updated the 1984 NINCDS-ADRDA criteria for higher specificity and sensitivity in the diagnosis of Alzheimer’s disease. The newly proposed criteria include probable and possible AD dementia for the use in clinical settings and probable or possible AD dementia with pathophysiological evidence for research purposes, in addition to clinical biomarkers. There are two categories of Alzheimer’s disease biomarkers: (a) markers of brain amyloid such as positron emission tomography (PET) and cerebrospinal fluid (CSF), and (b) markers of neuronal injury like cerebrospinal fluid tau, fluorodeoxyglucose (FDG) for metabolic activity, and magnetic resonance imaging (MRI) for atrophy measurement [13,14,15].

## 3. Alzheimer’s Disease’s Neuropathology

There are two types of neuropathological changes in AD which provide evidence about disease progress and symptoms and include: (1) positive lesions (due to accumulation), which are characterized by the accumulation of neurofibrillary tangles, amyloid plaques, dystrophic neurites, neuropil threads, and other deposits found in the brains of AD patients. In addition to (2) negative lesions (due to losses), that are characterized by large atrophy due to a neural, neuropil, and synaptic loss. Besides, other factors can cause neurodegeneration such as neuroinflammation, oxidative stress, and injury of cholinergic neurons [16,17,18].

### 3.1. Senile Plaques (SP)

The senile plaques are extracellular deposits of beta-amyloid protein (Aβ) with different morphological forms, including neuritic, diffuse, dense-cored, or classic and compact type plaques. Proteolytic cleavage enzymes such as β-secretase and γ-secretase are responsible for the biosynthesis of Aβ deposits from the transmembrane amyloid precursor protein (APP) [19,20,21]. These enzymes cleave APP into several amino acid fragments: 43, 45, 46, 48, 49, and 51 amino acids, which reach the final forms Aβ40 and Aβ42. There are several types of Aβ monomers, including large and insoluble amyloid fibrils which can accumulate to form amyloid plaques and soluble oligomers that can spread throughout the brain. Aβ plays a major role in neurotoxicity and neural function, therefore, accumulation of denser plaques in the hippocampus, amygdala, and cerebral cortex can cause stimulation of astrocytes and microglia, damage to axons, dendrites, and loss of synapses, in addition to cognitive impairments [21,22,23].

### 3.2. Neurofibrillary Tangles (NFTs)

NFT are abnormal filaments of the hyperphosphorylated tau protein that in some stages can be twisted around each other to form paired helical filament (PHF) and accumulate in neuralperikaryal cytoplasm, axons, and dendrites, which cause a loss of cytoskeletal microtubules and tubulin-associated proteins. The hyperphosphorylated tau protein is the major constituent of NFTs in the brains of AD patients, and its evolution can reflect NFTs morphological stages, which include: (1) pre-tangle phase, one type of NFT, where phosphorylated tau proteins are accumulated in the somatodendritic compartment without the formation of PHF, (2) mature NFTs, which are characterized by filament aggregation of tau protein with the displacement of the nucleus to the periphery part of the soma, and (3) the extracellular tangles, or the ghost NFTs stage, that results from a neuronal loss due to large amounts of filamentous tau protein with partial resistance to proteolysis [24,25].

### 3.3. Synaptic Loss

A synaptic damage in the neocortex and limbic system causes memory impairment and generally is observed at the early stages of AD. Synaptic loss mechanisms involve defects in axonal transport, mitochondrial damage, oxidative stress, and other processes that can contribute to small fractions, like the accumulation of Aβ and tau at the synaptic sites. These processes eventually lead to a loss of dendritic spines, pre-synaptic terminals, and axonal dystrophy [26]. Synaptic proteins serve as biomarkers for the detection of synapses loss, and severity, such as neurogranin, a postsynaptic neuronal protein, visinin-like protein-1 (VILIP-1), and synaptotagmin-1 [27,28].

## 4. The Stages of Alzheimer’s Disease

The clinical phases of Alzheimer’s disease can be classified into (1) pre-clinical or the pre-symptomatic stage, which can last for several years or more. This stage is characterized by mild memory loss and early pathological changes in cortex and hippocampus, with no functional impairment in the daily activities and absence of clinical signs and symptoms of AD [1,29,30]. (2) The mild or early stage of AD, where several symptoms start to appear in patients, such as a trouble in the daily life of the patient with a loss of concentration and memory, disorientation of place and time, a change in the mood, and a development of depression [30,31]. (3) Moderate AD stage, in which the disease spreads to cerebral cortex areas that results in an increased memory loss with trouble recognizing family and friends, a loss of impulse control, and difficulty in reading, writing, and speaking [30]. (4) Severe AD or late-stage, which involves the spread of the disease to the entire cortex area with a severe accumulation of neuritic plaques and neurofibrillary tangles, resulting in a progressive functional and cognitive impairment where the patients cannot recognize their family at all and may become bedridden with difficulties in swallowing and urination, and eventually leading to the patient’s death due to these complications [1,32].

## 5. Causes and Risk Factors of Alzheimer’s Disease

**Figure 2:**
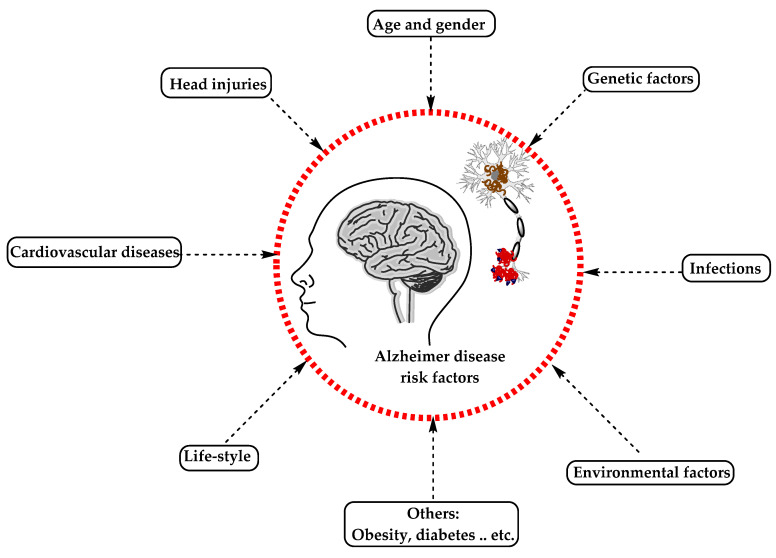
The risk factors for Alzheimer’s disease.

AD has been considered a multifactorial disease associated with several risk factors (Figure 2) such as increasing age, genetic factors, head injuries, vascular diseases, infections, and environmental factors (heavy metals, trace metals, and others). The underlying cause of pathological changes in Alzheimer’s disease (Aβ, NFTs, and synaptic loss) is still unknown. Several hypotheses were proposed as a cause for AD but two of them are believed to be the main cause: some believe that an impairment in the cholinergic function is a critical risk factor for AD, while others suggest that alteration in amyloid β-protein production and processing is the main initiating factor. However, at present, there is no accepted theory for explaining the AD pathogenesis [33,34].

### 5.1. Alzheimer’s Disease Hypotheses

#### 5.1.1. Cholinergic Hypothesis

**Figure 3:**
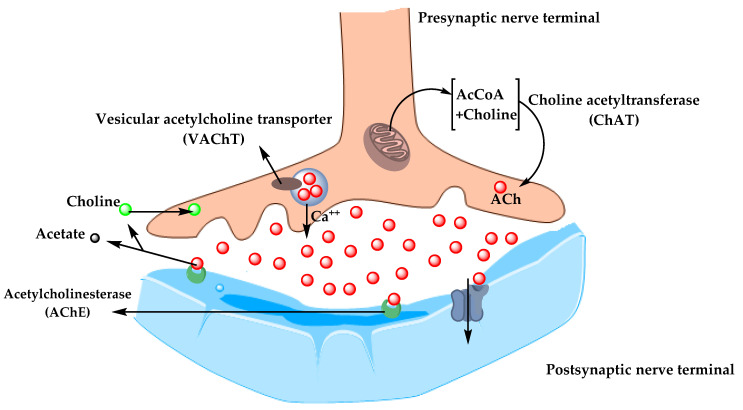
The pathway for the synthesis and transportation of acetylcholine between presynaptic and postsynaptic nerve terminals.

In the 1970s, neocortical and presynaptic cholinergic deficits were reported to be related to the enzyme choline acetyltransferase (ChAT), which is responsible for the synthesis of acetylcholine (ACh). Due to the essential role of ACh in cognitive function, a cholinergic hypothesis of AD was proposed. ACh is synthesized in the cytoplasm of cholinergic neurons from choline and acetyl-coenzyme A by the ChAT enzyme and transported to the synaptic vesicles by vesicular acetylcholine transporter (VAChT) (Figure 3). In the brain, ACh is involved in several physiological processes such as memory, attention, sensory information, learning, and other critical functions. Degeneration of the cholinergic neurons was found to take place in AD and to cause alternation in cognitive function and memory loss. *Β*-amyloid is believed to affect cholinergic neurotransmission and to cause a reduction in the choline uptake and a release of ACh. Studies demonstrated that cholinergic synaptic loss and amyloid fibril formation are related to Aβ oligomers’ neurotoxicity and to interactions between AChE and Aβ peptide. Additional factors also contribute to the progression of AD, such as a reduction in nicotinic and muscarinic (M2) Ach receptors, located on presynaptic cholinergic terminals, and the deficit in excitatory amino acid (EAA) neurotransmission, where glutamate concentration and D-aspartate uptake are significantly reduced in many cortical areas in AD brains. This is in addition to the use of cholinergic receptor antagonists such as scopolamine, which was found to induce amnesia. This effect can be reversed by using compounds that activate acetylcholine formation [35,36,37].

As a result, the cholinergic hypothesis is based on three concepts: reduced presynaptic cholinergic markers in the cerebral cortex, severe neurodegeneration of nucleus basalis of Meynert (NBM) in the basal forebrain, which is the source of cortical cholinergic innervation, and the role of cholinergic antagonists in memory decline compared to the agonists, which have the opposite effect [38].

#### 5.1.2. Amyloid Hypothesis

For decades, it was recognized that abnormal deposition of β-sheets in the central nervous system has a strong correlation with dementia, which led to the concept of the amyloid hypothesis. However, it was found that the amyloid plaques (AP) also deposit in normal healthy brains with aging, which raised the question of whether AP deposition is responsible for AD onset or not? Therefore, in the recent years, alternative hypotheses were proposed for the non-inherited form of AD (NIAD), but at present, the amyloid hypothesis remains the most accepted pathological mechanism for inherited AD (IAD). The amyloid hypothesis suggests that the degradation of Aβ, derived from APP by β- and γ-secretase, is decreased by age or pathological conditions, which leads to the accumulation of Aβ peptides (Aβ40 and Aβ42). Increasing the ratio of Aβ42/Aβ40 induces Aβ amyloid fibril formation, resulting in neurotoxicity and tau pathology induction, and consequently, leading to neuronal cell death and neurodegeneration. AD risk factors and mutations of several genes like APP, PSEN1, and PSEN2 were found to affect Aβ catabolism and anabolism, which rapidly cause an accumulation of Aβ and fast progression of neurodegeneration [39,40,41].

### 5.2. Alzheimer’s Disease Risk Factors

#### 5.2.1. Aging

The most important risk factor in AD is aging. Younger individuals rarely have this disease, and most AD cases have a late onset that starts after 65 years of age [42]. Aging is a complex and irreversible process that occurs through multiple organs and cell systems with a reduction in the brain volume and weight, a loss of synapses, and ventricles’ enlargement in specific areas accompanied by SP deposition and NFT. Moreover, several conditions might emerge during aging such as glucose hypometabolism, cholesterol dyshomeostasis, mitochondria dysfunction, depression, and cognitive decline. These changes also appear in normal aging, which makes it difficult to distinguish the cases in early AD [43,44]. AD can be divided based on age of onset into early-onset AD (EOAD), the rare form with around 1–6% of cases, in which most of them are familial AD characterized by having more than one member in more than one generation with AD, and ranges from 30–60 or 65 years. The second type is the late-onset AD (LOAD), which is more common with age of onset above 65 years. Both types may occur in people who have a family with a positive history of AD and families with a late-onset disease [45].

#### 5.2.2. Genetics

Genetic factors were discovered over the years and were found to play a major role in the development of AD. 70% of the AD cases were related to genetic factors: most cases of EOAD are inherited in an autosomal dominant pattern and mutations in the dominant genes such as *Amyloid precursor protein (APP)*, Presenilin-1 (PSEN-1), *Presenilin-2 (PSEN-2)*, and apolipoprotein E (ApoE) are associated with AD [46,47].

Herein, we discuss the strong genetic risk factors in AD.

- Amyloid Precursor Protein (APP)

APP is a type I transmembrane protein cleaved by α-, β-, and γ-secretase to release Aβ and other proteins and is encoded by the APP gene on chromosome 21. Thirty mutations have been found in the APP gene in which twenty-five of them are related to AD and cause an accumulation of Aβ with elevated amounts. Meanwhile, there is one protective mutation, A673T, which protects against AD by decreasing Aβ, Aβ40, and Aβ42 secretion [48,49]. All mutations surround the secretase cleavage site, for example, the KM670/671NL mutation in mouse models has shown an increasing level of amyloid plaques in the hippocampus and cortex with no NFTs. A673V, D678H, D678N, E682K, and K687N mutations have shown cortical atrophy, whereas E682K has shown hippocampal atrophy. Neuropathological reports for the A673V mutation demonstrated a presence of NFTs and Aβ, activation of microglia and astrocytes, and neuronal loss, compared to the rest of the mentioned mutations, which show no change in the intracellular Aβ according to neuropathological reports [48,50]. Other mutations such as T714I, V715A, V715M, V717I, V717L, L723P, K724N, and I716V affect the γ-secretase cleavage site and cause an increase in the Aβ42/Aβ40 ratio, while E693G, E693K, D694N, and A692G mutations affect the α-secretase cleavage site and cause polymorphic aggregates with the ability to disrupt bilayer integrity. Also, the E693delta is a deletion mutation that enhances the formation of synaptotoxic Aβ [51,52].

- Presenilin-1 (PSEN-1) and Presenilin-2 (PSEN-2)

*PSEN1* and *PSEN2* genes are also the autosomal dominant form of EOAD located on chromosomes 14 and 1, respectively. PSEN-2 and PSEN-1 are homologous, with 67% similarity, with a difference in the *N*-terminus and the hydrophilic region. Mutation in *PSEN1* gene is more common, with more than 200 mutations, while a rare form with less than 40 mutations was identified in the *PSEN2* gene [53,54].

PSEN1 is a core protein that activates the γ-secretase complex and plays an important role in the production of Aβ from APP. Knockout studies of PSEN1 showed synaptic dysfunction and memory impairment in mice, which indicate its essential role in maintaining memory and neurons [51]. *PSEN1* mutations are simple ones which include single amino acid substitution, and severe mutation can result from the substitutions of two amino acids [55]. Mutations in the *PSEN1* gene increase the ratio of Aβ42/Aβ40 by decreasing Aβ40 levels. The results obtained by Sun et al. study demonstrated that C410Y or L435F mutations in *PSEN1* knock-in mice increased the Aβ42/Aβ40 ratio due to a greater reduction in Aβ40 [56].

In contrast, PSEN-2 mutations are rare and play a minor role in Aβ production. Any mutation in *PSEN-2* might have a severe effect on the Aβ 42/40 ratio, causing familial AD in the presence of normal *PSEN-1* alleles. Some of the *PSEN-2* mutations cause a significant increase in γ-secretase activity with an elevation in the Aβ-42 and Aβ 42/40 ratio level, such as N141I, T122P, M239V, and M239I, while others are rare polymorphisms and have no effect on Aβ-42, -40, and Aβ 42/40 ratio levels and are not considered as pathogenic mutations [53,57].

- Apolipoprotein E (ApoE)

ApoE protein is a glycoprotein expressed highly in the liver and brain astrocytes and some microglia and serves as a receptor-mediated endocytosis ligand for lipoprotein particles like cholesterol, which is essential for myelin production and normal brain function. The ApoE gene located on chromosome 19 has three isoforms, ApoE2, ApoE3, and ApoE4, due to single-nucleotide polymorphisms (SNPs) which cause changes in the coding sequence. The ApoEε4 allele is a strong risk factor for both EOAD and LOAD compared to ApoEε2 and ApoEε3 alleles that are associated with a lower risk and protective effect, respectively [58]. ApoEε4 plays an important role in Aβ deposition as a senile plaque and causes cerebral amyloid angiopathy (CAA), which is known as a marker for AD [59]. ApoEε4 was also shown to be associated with vascular damage in the brain, which leads to AD pathogenesis [60].

- ATP Binding Cassette Transporter A1 (ABCA1)

Adenosine triphosphate (ATP)-binding cassette transporter A1 (ABCA1) is part of a large ABC transporters family that regulate cholesterol efflux in the circulation, like apolipoproteins-AI (ApoAI), and into the brain, like ApoE. In addition, ABCA1 maintains the stability of ApoE lipidation and serves as a mediator for high-density lipoprotein (HDL) generation, which reflects its role in atherosclerosis and cardiovascular diseases. Studies on the AD mice model showed that ABCA1 deficiency increases amyloid plaques and eliminates the lipidation of ApoE [61]. In humans, a mutation in ABCA1 results in Tangier disease, which is characterized by low levels of high-density lipoprotein (HDL) and ApoAI in plasma, accumulation of cholesterol in tissues, and AD pathogenesis [62].

- Clusterin Gene (CLU) and Bridging Integrator 1 (*BIN1*)

In contrast to *PSEN1*, *PSEN2*, and *APP* mutations, which result in familial or EOAD, clusterin (*CLU)* and Bridging Integrator 1 (*BIN1*) genes are novel risk factors for LOAD. In 2009, Genome-Wide Association Studies (GWAS) identified the CLU gene located on chromosome 8, which is upregulated in the cortex and hippocampus of AD brains, in addition to AD cerebrospinal fluid (CSF) and plasma, which make the CLU a promising biomarker for AD. The CLU may play a protective role by interacting with Aβ and promoting its clearance, or a neurotoxic role by reducing Aβ clearance. The Aβ ratio values determine whether the CLU role is neuroprotective or neurotoxic [63].

*BIN1* is a Bin-Amphiphysin-Rvs (BAR) adaptor protein that is involved in the production of membrane curvature and other endocytosis cellular functions. *BIN1* has several isoforms: some are found in the brain, where they interact with different proteins such as clathrin, synaptojanin, and amphiphysin 1, and others in which they regulate synaptic vesicle endocytosis. Recently, BIN1 was recognized as the second most important risk factor for LOAD after ApoE, where it plays a role in Aβ production and as a tau and NFT pathology modulator [64,65].

- Evolutionarily Conserved Signaling Intermediate in Toll pathway (ECSIT)

A significant accumulation of Aβ in AD brains increases protein oxidation, which reflects the critical role of mitochondria in Aβ cytotoxicity and AD pathogenesis. Evolutionarily conserved signaling intermediate in Toll pathway (ECSIT) gene is located on chromosome 19 and is associated with increasing the risk of AD. ECSIT encodes the adapting protein that functions as a cytoplasmic and signaling protein and is responsible for stabilizing the mitochondrial respiratory complex. Moreover, the adaptor protein is involved in the activation of nuclear factor (NF)-κB, interferon regulatory factors (IRFs), and activating protein-1. Also, it is involved in coupling immune toll-like receptor (TLR), homeostatic bone morphogenetic pathway (BMP), and transforming growth factor-beta (TGF-b) pathways [66,67].

ECSIT interacts with mitochondrial proteins such as Lon protease homolog (LONP1) and glutaryl-CoA dehydrogenase (GCDH), which are involved in intra-mitochondrial proteolysis and redox signaling respectively, followed by interactions with AD seed nitric oxide synthase (NOS3). Moreover, studies have shown certain interactions of *ECSIT* with the AD genes *ApoE*, *PSEN-1*, and *PSEN-2*. These interactions support the role of *ECSIT* as a molecular link in oxidative stress, inflammation, and mitochondrial dysfunction in AD [66,68].

- Estrogen Receptor Gene (ESR)

AD affects both women and men, but nearly two-thirds of AD cases are women. Several studies have shown that women with AD experience worse mental deterioration than men. Additionally, on the genetic level, some genes’ variation, like the ApoE4 allele, significantly increases AD risk in women compared to men. Other studies documented that AD risk in women is associated with the loss of ovarian hormones during menopause due to the fact that estrogen regulates several activities in the brain, such as neurotransmission, neural development, survival, protection against oxidative stress, reduction of Aβ peptide levels, and attenuation of tau hyperphosphorylation. The estrogen activity is mediated through estrogen receptors (ERs) (intracellular, transmembrane, and membrane-bound ERs). The two major subtypes of these receptors are ERα and Erβ, which are encoded by two distinct genes and are located on chromosome 6 and 14, respectively. ERα receptor is found in the hypothalamus and amygdala, whereas ERβ receptors are in the hippocampus and cortex. Single nucleotide polymorphisms (SNPs) in ERβ and ERα genes may affect exogenous estrogen in older women and influence cognitive aging. PvuII (rs9340799) and Xbal (rs223493) are examples of SNPs found in ERα and are associated with AD and cognitive impairment. Also, several SNPs in ERβ have been proven to increase the risk of AD in women [69,70,71,72].

- Other Genes

Other genes’ polymorphism associated with increasing the risk of AD include vitamin D receptor (VDR) gene polymorphism, which affects the affinity of vitamin D to its receptor and may cause neurodegenerative diseases and neuronal damage [73]. Moreover, epigenetic factors like DNA methylation, histone, and chromatin modifications were demonstrated to be involved in AD [33,74].

#### 5.2.3. Environmental Factors

Aging and genetic risk factors cannot explain all cases of AD. Environmental risk factors including air pollution, diet, metals, infections, and many others may induce oxidative stress and inflammation and increase the risk for developing AD. Herein, we report the most important environmental factors and their relationships with AD [75,76].

- Air Pollution

The air pollution is characterized by modifying the nature of the atmosphere through the introduction of chemical, physical, or biological pollutants. It is associated with respiratory and cardiovascular diseases and recently, its association with AD was documented. Six air pollutants have been defined by National Ambient Air Quality Standards (NAAQSs) in the USA as a threat to human health, including ozone (O_3_), nitrogen oxides (NO_x_), carbon monoxide (CO), particulate matter (PM), sulfur dioxide (SO_2_), and lead. Studies on animals and cellular models have shown that an exposure to high levels of air pollution can result in a damage to the olfactory mucosa and bulb, in addition to the frontal cortex region, similar to that observed in AD. In individuals exposed to air pollutants, there is a link between oxidative stress, neuroinflammation, and neurodegeneration, with the presence of hyper-phosphorylated tau and Aβ plaques in the frontal cortex. The air pollution can cause an increase in Aβ_42_ formation, accumulation, and impaired cognitive function [77,78].

- Diet

In recent years, the number of studies on the role of nutrition in AD have been increased. Several dietary supplements such as antioxidants, vitamins, polyphenols, and fish were reported to decrease the risk of AD, whereas saturated fatty acids and high-calorie intake were associated with increasing the risk of AD [79]. The food processing causes degradation of heat-sensitive micronutrients (e.g., vitamin C and folates), loss of large amounts of water, and formation of toxic secondary products (advanced glycation end products, AGEs) from non-enzymatic glycation of free amino groups in proteins, lipids, and nucleic acids. The toxic effect of AGEs is referred to as their ability to induce oxidative stress and inflammation by modifying the structure and function of the cell surface receptors and body proteins. Different studies demonstrated that elevated AGEs serum level is associated with cognitive decline and progression of AD. The AGE receptor (RAGE) is located in different places within the body, including microglia and astrocytes, and was established to be overexpressed in the brain of AD patients and serve as a transporter and a cell surface receptor for Aβ [80]. Malnutrition is another risk factor for AD. Deficiency in nutrients such as folate, vitamin B12, and vitamin D may cause a decrease in cognitive function, in addition to the fact that patients with AD suffer from problems associated with eating and swallowing, which may increase the risk of malnutrition [81].

- Metals

Metals are found in nature and biological systems and can be divided into bio-metals that have a physiological function in living organisms (e.g., copper, zinc, and iron), and toxicological metals which do not possess any biological function (e.g., aluminum and lead) [82]. Aluminum is used significantly in the industries such as processed foods, cosmetics, medical preparations, medicines, and others. In the body, aluminum is bound to plasma transferrin and to citrate molecules that can mediate the transfer of aluminum to the brain. Studies demonstrated that Al accumulates in the cortex, hippocampus, and cerebellum areas, where it interacts with proteins and causes misfolding, aggregation, and phosphorylation of highly phosphorylated proteins like tau protein, characteristic of AD [83]. Lead competes with the binding site of bio-metals like calcium and can cross the blood–brain barrier (BBB) rapidly, where it can modify neural differentiation and synaptogenesis and cause severe damage. Studies revealed that an acute exposure to lead was associated with AD and caused an increase of β-secretase expression and Aβ accumulation. Cadmium is a carcinogenic water-soluble metal that can cross the BBB and cause neurological diseases like AD. Results have demonstrated that Cadmium ions are involved in the aggregation of Aβ plaques and the self-aggregation of tau in the AD brain. The data accumulated on metals support the notion that they are among the risk factors involved in the development of AD [84].

- Infections

Chronic infections to the central nervous system (CNS) can cause an accumulation of Aβ plaques and NFT, therefore, they are included among the risk factors in AD. Studies by Dr. Itzhaki showed that the DNA of herpes simplex virus (HSV-1) was found in patients with ApoE-ε4 allele carriers, which explains the high risk for developing AD. HSV-1 can replicate in the brain, which can result in the activation of the inflammatory response and an increase in Aβ deposition, resulting in damage to neurons and gradual development of AD. On the other hand, the study results by Miklossy and Balin’s have revealed the role of chronic bacterial infections in AD. For example, syphilitic dementia caused by spirochete bacteria (*Treponema pallidum*), which are accumulated in the cerebral cortex, produced lesions similar to neurofibrillary tangles, which led to devastating neurodegenerative disorders. Besides, *Chlamydia pneumonia* bacterium can trigger late-onset AD by activation of astrocyte and cytotoxic microglia, disrupt calcium regulation and apoptosis, resulting in deterioration of cognitive function, and increase the risk of AD [85,86,87].

#### 5.2.4. Medical Factors

Several risk factors are related to the development of Alzheimer’s disease. Adding to this list, older people with AD usually have medical conditions such as cardiovascular disease (CVD), obesity, diabetes, and others. All of these conditions are associated with increased risk of AD [88,89].

- Cardiovascular Disease (CVDs)

CVDs are recognized as an important risk factor for AD, such as the stroke that is associated with increased risk of dementia due to a neural tissue loss, which enhances degenerative effect and influences amyloid and tau pathology. Atrial fibrillation also causes embolisms which leads to stroke and a decrease in memory and cognitive functions. Moreover, heart failure affects the pumping function of the heart and results in insufficient blood supply to the body and hypo-perfusion of the brain that leads to hypoxia and neural damage. The coronary heart disease’s hypothesis indicates that atherosclerosis, peripheral artery disease, hypo-perfusion, and emboli are all related to increased risk of AD. Hypertension is associated with thickening of vessel walls and narrowing of the lumen which reduce the cerebral blood flow, and in chronic cases, it may cause cerebral edema, which all participate as risk factors for AD and CVD. The CVD is a modifiable risk factor and by focusing on its relationship with AD, a pathway to prevent and delay the disease can be obtained [89,90].

- Obesity and Diabetes

Obesity is a term used for too much body fat in individuals due to consuming more calories than they burn and can be calculated by using the body mass index (BMI). Increasing the body fat is associated with a decreased brain blood supply which promotes brain ischemia, memory loss, and vascular dementia. The obesity, unhealthy diet, and other factors can cause impaired glucose tolerance (IGT) or diabetes, which is characterized by hyperglycemia that affects peripheral tissues and blood vessels. Chronic hyperglycemia can induce cognitive impairment as a result of increasing amyloid-beta accumulation, oxidative stress, mitochondrial dysfunction, and neuroinflammation. Obesity is characterized by increasing pro-inflammatory cytokines secretions from adipose tissue, which stimulate macrophages and lymphocytes and eventually lead to local and systemic inflammation. This inflammation promotes insulin resistance, hyperinsulinemia, and as a consequence, hyperglycemia. Obesity is a well-known risk factor for type 2 diabetes, CVDs, and cancer, which are identified as risk factors for dementia and AD. The brain inflammation causes an increase in microglia and results in reduced synaptic plasticity and impaired neurogenesis. Microglia can affect insulin receptor substrate 1 (IRS-1) and block intracellular insulin signaling, which has an important role in neural health. Therefore, alteration in insulin action can result in Aβ accumulation and reduce the tau protein degradation associated with AD [91,92,93,94].

## 6. Treatment

Currently, Alzheimer’s disease cases worldwide are reported to be around 24 million, and in 2050, the total number of people with dementia is estimated to increase 4 times. Even though AD is a public health issue, as of now, there is only two classes of drugs approved to treat AD, including inhibitors to cholinesterase enzyme (naturally derived, synthetic and hybrid analogues) and antagonists to *N*-methyl d-aspartate (NMDA). Several physiological processes in AD destroy Ach-producing cells which reduce cholinergic transmission through the brain. Acetylcholinesterase inhibitors (AChEIs), which are classified as reversible, irreversible, and pseudo-reversible, act by blocking cholinesterase enzymes (AChE and butyrylcholinesterase (BChE)) from breaking down ACh, which results in increasing ACh levels in the synaptic cleft [95,96,97]. On the other hand, overactivation of NMDAR leads to increasing levels of influxed Ca^2+^, which promotes cell death and synaptic dysfunction. NMDAR antagonist prevents overactivation of NMDAR glutamate receptor and hence, Ca^2+^ influx, and restores its normal activity. Despite the therapeutic effect of these two classes, they are effective only in treating the symptoms of AD, but do not cure or prevent the disease [98,99]. Unfortunately, only a few clinical trials on AD have been launched in the last decade and their outcome was a big failure. Several mechanisms have been proposed to understand AD pathology in order to modify its pathway and develop successful treatments, which include abnormal tau protein metabolism, β-amyloid, inflammatory response, and cholinergic and free radical damage [30,100]. On the other hand, most AD modifiable risk factors such as cardiovascular or lifestyle habits can be prevented without medical intervention. Studies showed that physical activity can improve the brain health and reduce AD by activating the brain vascularization, plasticity, neurogenesis, and reducing inflammation by decreasing Aβ production, which all result in improving cognitive function in older people. Moreover, the Mediterranean diet (MD), intellectual activity, and higher education all may reduce the progression of AD and memory loss and increase the brain capacity and cognitive functions. Several studies revealed that multi-domain intervention which includes lifestyle (diet, exercise, and cognitive training), depression of AD symptoms, and controlling cardiovascular risk factors, can increase or maintain cognitive function and prevent new cases of AD in older people [101]. Herein, we summarize the currently available drugs and theories for the development of new therapies for AD.

### 6.1. Symptomatic Treatment of AD

#### 6.1.1. Cholinesterase Inhibitors

**Figure 4:**
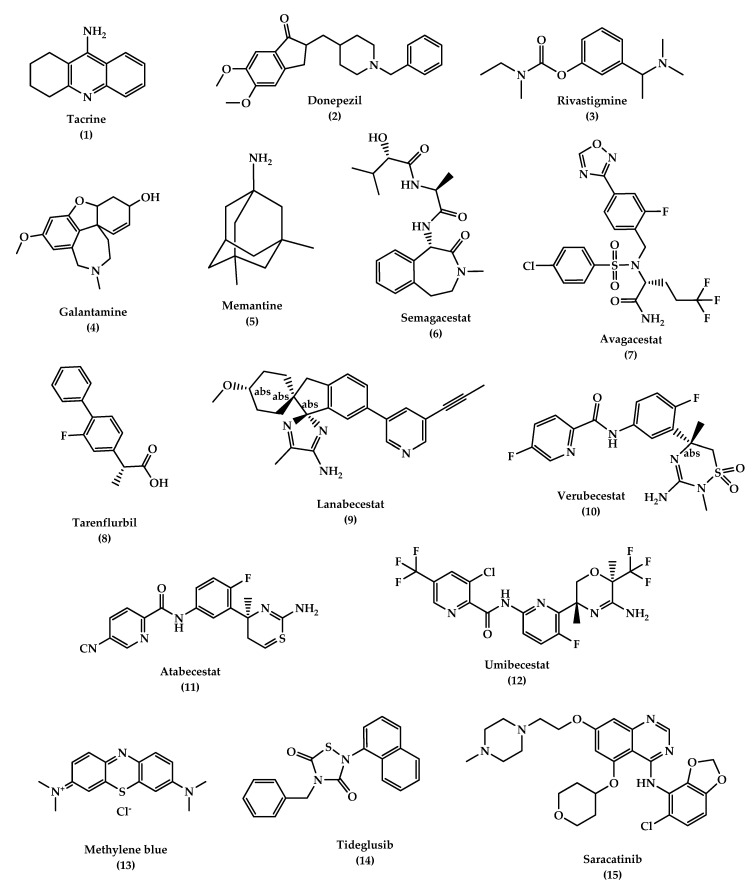
The chemical structures of approved drugs for symptomatic treatment of AD (tacrine **1**, donepezil **2**, rivastigmine **3**, galantamine **4**, and memantine **5**) and disease-modifying compounds that entered clinical trials (semagacestat **6**, avagacestat **7**, tarenflurbil **8**, lanabecestat **9**, verubecestat **10**, atabecestat **11**, umibecestat **12**, methylene blue **13**, tideglusib **14**, and saracatinibin **15**).

According to the cholinergic hypothesis, AD is due to the reduction in acetylcholine (ACh) biosynthesis. Increasing cholinergic levels by inhibiting acetylcholinesterase (AChE) is considered one of the therapeutic strategies that increases cognitive and neural cell function. AChEIs are used to inhibit acetylcholine degradation in the synapses, which results in continuous accumulation of ACh and activation of cholinergic receptors. Tacrine (tetrahydroaminoacridine) (**1,** Figure 4) was the first FDA (Food and Drug Administration)-approved cholinesterase inhibitor drug for the treatment of AD, which acts by increasing ACh in muscarinic neurons, but it exited the market immediately after its introduction due to a high incidence of side effects like hepatotoxicity and a lack of benefits, which was observed in several trials. Later on, several AChEIs were introduced, such as donepezil (**2**, Figure 4), rivastigmine (**3**, Figure 4), and galantamine (**4**, Figure 4), and are currently in use for the symptomatic treatment of AD [34,97,102,103]. Another strategy that may help in the treatment of AD is increasing choline reuptake and as a result, increasing acetylcholine synthesis at the presynaptic terminals. This can be achieved by targeting choline transporter (CHT1) which is responsible for supplying choline for the synthesis of ACh. Developing drugs that are capable of increasing CHT1 at the plasma membrane may become the future therapy of AD [36].

- Donepezil

Donepezil (**2**, Figure 4) is an indanonebenzylpiperidine derivative and a second generation of AChEIs and is considered the leading drug for AD treatment. Donepezil binds to acetylcholinesterase reversibly and inhibits acetylcholine hydrolysis, which leads to a higher concentration of ACh at the synapses. The drug is well-tolerated with mild and transient cholinergic side effects which are related to the gastrointestinal and nervous systems. It should be noted that donepezil is used to treat symptoms of AD such as improving cognition and behavior without altering the AD progression [104,105,106].

- Rivastigmine

Rivastigmine (**3**, Figure 4) is a pseudo irreversible inhibitor of AChE and butyrylcholinesterase (BuChE) that acts by binding to the two active sites of AChE (anionic and estearic sites), which results in preventing ACh metabolism. BuChE is found mostly in glial cells with only 10% of AChE activity in the normal brain, whereas in the AD brain, its activity is increased to 40–90%, while ACh activity is reduced simultaneously, which suggests that BuChE action may indicate a moderate to severe dementia. Rivastigmine dissociates more slowly than AChE, which is why it is called a pseudo-irreversible, and it undergoes metabolism at the synapse by AChE and BuChE. The drug is used in mild to moderate AD cases. It improves cognitive functions and daily life activities. Oral administration of the drug is associated with adverse effects such as nausea, vomiting, dyspepsia, asthenia, anorexia, and weight loss. In many cases, these side effects are the main reason behind stopping taking the medicine, however, they can be settled down in time and consequently, the drug becomes more tolerated. Rivastigmine can be delivered by transdermal patches for controlled and continuous delivery of the drug through the skin, with enhanced tolerability and caregiver satisfaction. Also, the patches can deliver a lower dosage compared to pills, which results in reduced side effects. Most AD patients suffer from memory loss and swallowing problems which affect their compliance in administering oral drugs at regular intervals. Therefore, the use of transdermal patches is the most appropriate method for delivering the drug in AD patients [107,108,109,110].

- Galantamine (GAL)

Galantamine (**4**, Figure 4) is considered a standard first-line drug for mild to moderate AD cases. GAL is a selective tertiary isoquinoline alkaloid with a dual mechanism of action in which it acts as a competitive inhibitor of AChE and can bind allosterically to the α-subunit of nicotinic acetylcholine receptors and activate them. GAL can improve behavioral symptoms, daily life activities, and cognitive performance with good efficacy and tolerability, similar to other AChE inhibitors. Several delivery systems were developed to improve the drug delivery to the brain: Wahba et al. attached GAL to ceria-containing hydroxyapatite particles for selective delivery of the drug to the affected regions in the brain. Misra et al. and Fornaguera et al. used solid-lipid nanoparticles and nano-emulsification approaches respectively, to carry GAL hydrobromide. The results of these studies demonstrated a promising strategy for safe delivery of the drug. Hanafy et al. developed nasal GAL hydrobromide/chitosan complex nanoparticles which showed good pharmacological efficacy, while Woo et al. utilized the patch system as a carrier for a controlled release dosage form of the drug [111,112,113,114].

#### 6.1.2. *N*-methyl d-aspartate (NMDA) Antagonists

NMDAR is believed to have a dominant role in the pathophysiology of AD. NMDAR stimulation results in Ca^2+^ influx which activates signal transduction and as a consequence, it triggers gene transcription essential for the formation of a long-term potentiation (LTP), which is important for synaptic neurotransmission, plasticity, and memory formation. Over-activation of NMDARs causes an abnormal level of Ca^2+^ signaling and overstimulation of glutamate, which is the primary excitatory amino acid in the CNS, which results in excitotoxicity, synaptic dysfunction, neuronal cell death, and a decline in cognitive functions. Several NMDAR uncompetitive antagonists have been developed and entered clinical trials, however, most of them failed due to low efficacy and side effects. Memantine (**5**, Figure 4) is the only approved drug in this category to treat moderate to severe AD; in addition, other NMDAR uncompetitive antagonist compounds are being developed, such as RL-208 (3,4,8,9-tetramethyltetracyclo [4.4.0.0^3,9^.0^4,8^]dec-1-yl)methylamine hydrochloride), a polycyclic amine compound that may possess a promising therapeutic effect in age-related cognitive problems and AD [115,116,117].

- Memantine

Memantine (**5**, Figure 4) is a low-affinity uncompetitive antagonist of the NMDAR, a subtype of glutamate receptor that prevents over-activation of the glutaminergic system involved in the neurotoxicity in AD cases. Memantine is used for the treatment of moderate to severe AD alone or in combination with AChEI. The drug is safe and well-tolerated, it blocks the excitatory receptor without interfering with the normal synaptic transmission due to memantine’s low affinity, where it is displaced rapidly from NMDAR by high concentrations of glutamate, thus avoiding a prolonged blockage. The latter is associated with high side effects, especially on learning and memory [99,118].

### 6.2. Promising Future Therapies

#### 6.2.1. Disease-Modifying Therapeutics (DMT)

Disease-modifying treatment or therapy (DMT) alter the progression of AD by working on several pathophysiological mechanisms. This is in contrast to symptomatic therapy which works on improving the cognitive functions and decreasing symptoms such as depression or delusions without affecting or modifying the disease. DMTs, either immunotherapies or small molecules, are administrated orally and are being developed to prevent AD or decrease its progression. Several DMTs have been developed and entered the clinical trials, such as AN-1792, a synthetic Aβ peptide (human Aβ_1–42_ peptide of 42-amino acids with the immune adjuvant QS-21) and the first active immunotherapy for AD which entered phase II clinical trials and discontinued due to a meningoencephalitis side effect in 6% of the patients. Other drugs were also developed and failed in the clinical trials, including the anti-Aβ antibody (solanezumab and bapineuzumab), γ-Secretase inhibitors (semagacestat **6**, avagacestat **7**, and tarenflurbil **8**) (Figure 4) and β-secretase inhibitors (BACE) (Lanabecestat **9,** verubecestat **10**, and atabecestat **11**) (Figure 4). DMTs failures are due to several factors, such as starting therapy too late, giving treatment for the wrong main target, use of inappropriate drug doses, and misunderstanding of the pathophysiology of AD. Several immunotherapies described in Table 1 have been developed over decades, including: CAD106, an active Aβ immunotherapy that induces Aβ antibodies in animal models and consists of multiple copies of Aβ1–6 peptide coupled to Qβ coat protein, a virus-like particle, and is still in clinical trials, and CNP520 (umibecestat, **12**) (Figure 4), a small molecule that inhibits beta-scretase-1 (BACE-1) and therefore inhibits Aβ production. CNP520 was found to reduce Aβ plaque deposition and Aβ levels in the brain and CSF in rats, dogs, and healthy adults ≥ 60 years old, and is still under clinical trials. Furthermore, aducanumab, gantenerumab, and crenezumab are all human Aβ monoclonal antibody that bind with high affinity to aggregated Aβ, and they are still under study in the clinical phases with other DMTs described in Table 1 [6,119,120,121,122,123,124].

Another class targeting the α-secretase enzyme was developed and has been considered as therapeutic agents. α-secretase modulators or activators stimulate the cleavage of APP. There is little knowledge about the activation pathway, but research assumes that it is promoted by the phosphatidylinositol 3-kinase (PI3K)/Akt pathway or by γ-aminobutyric acid (GABA) receptor signaling. Targeting these pathways may give potential therapeutic agents for AD [6].

In addition to the anti-amyloid agents, the tau aggregation inhibitors are another promising DMT. The tau is a biomarker for neurofibrillary tangles (NFT) in AD and naturally modulates microtubule stability, signaling pathways, and axonal transport. A modification in tau conformation results in toxic aggregation. Therefore, the prevention of tau aggregation becomes an interesting approach for drug discovery to reduce AD progression. Studies in mice have shown that tau oligomers cause mitochondrial damage, disruption of neuronal signaling, synaptic loss, and memory impairment. Disease-modifying therapeutics (DMT) like small molecules can be used to inhibit the initial step in the tau aggregation and thereby reduce its accumulation. Methylene blue (**13**, Figure 4) is a blue dye that inhibits the tau aggregation and entered phase II clinical trials to treat mild to moderate AD. Upon administration of the drug, the color of the urine becomes blue, which indicates a lack of binding, and because of that, the study was highly criticized. Other approaches suggest that an inhibition of specific kinases such as glycogen synthase kinase 3 (GSK3β) can inhibit tau hyperphosphorylation and block tau deposition. Examples of these entities include tideglusib (**14**, or NP-031112 (NP-12), Figure 4), a thiazolidinedione-derived compound, lithium, pyrazolopyridines, pyrazolopyrazines, sodium valproate, and others. Another protein kinase inhibitor is saracatinib (AZD0530) (**15**, Figure 4), which acts by inhibiting tyrosine kinase and has shown good results in improving memory in transgenic mice and is currently in phase II trials [125,126,127]. Davidowitz et al. utilized the hatu mouse model of tauopathy to study the efficacy of a lead small molecule in preventing tau accumulation. The study results demonstrated a significant reduction in tau levels and its phosphorylated form levels, which indicates the ability to inhibit the entire pathway of the tau aggregation by using an optimized lead compound [128].

#### 6.2.2. Chaperones

Protein misfolding caused by mutations or environmental factors results in aggregations that are toxic, and their accumulation causes neurodegenerative disorders like AD. Naturally, cells develop protein quality control (PQC) systems that inhibit protein misfolding before exerting their toxic effects. With age, this balance is altered and the misfolded shapes overwhelm the PQC system, which in turn activates the unfolded protein response (UPR) that stops the protein synthesis and increases chaperone production. Generally, the cells in humans have proteins that are responsible for other proteins to function and arrive to their destination in the cell. These proteins are called “chaperones”. Chaperones are involved in protein folding and improvement of the PQC system efficiency. Therefore, it is considered a promising candidate for treating neurodegenerative diseases. It can be classified into three groups: (1) molecular chaperones, which are proteins that assist other nonnative proteins in their folding or unfolding, like overexpression of heat shock proteins (Hsps) that serve as neuroprotective agents, (2) pharmacological chaperones, which are low molecular weight compounds (enzymes or receptor-ligand or selective binding molecules) that induce refolding of proteins, stabilize their structure, and restore their function, and (3) chemical chaperones, also low molecular weight compounds, which are divided into two groups, osmolytes and hydrophobic compounds. The members in these two groups have no specific mechanism of action and need high concentrations to exert their therapeutic effects [129].

- Heat Shock Proteins (Hsps)

The causes for most neurodegenerative diseases are protein misfolding and aggregation, which lead to cell death. The molecular chaperone can be intracellular, such as in the case of heat shock proteins (e.g., Hsp40, Hsp60, Hsp70, Hsp90, Hsp100, and Hsp110), and extracellular, such as clustering and alpha-macroglobulin. HSPs play an essential role in the protein folding process and protect cells from harmful stress-related events. There are two families of Hsps: (a) classic Hsps that possess an ATP-binding site with a molecular weight of 60 kD or more. This family includes Hsp100, Hsp90, Hsp70, and Hsp60, and (b) the small Hsps such as αB-crystalline, Hsp27, Hsp20, HspB8, and HspB2/B3 that lack ATP-binding site, with a molecular weight of 40 kD or less. These proteins can assist other Hsps in their refolding function. Failure of these mechanisms can lead to oxidative stress, mitochondrial dysfunction, and many other conditions that cause damage, a loss of neurons, and a progression of neurodegenerative diseases. Different HSPs can block the aggregation process of misfolded proteins, like amyloidogenic proteins (Aβ and tau), and promote their degradation [130,131].

1. Hsp60

**Figure 5:**
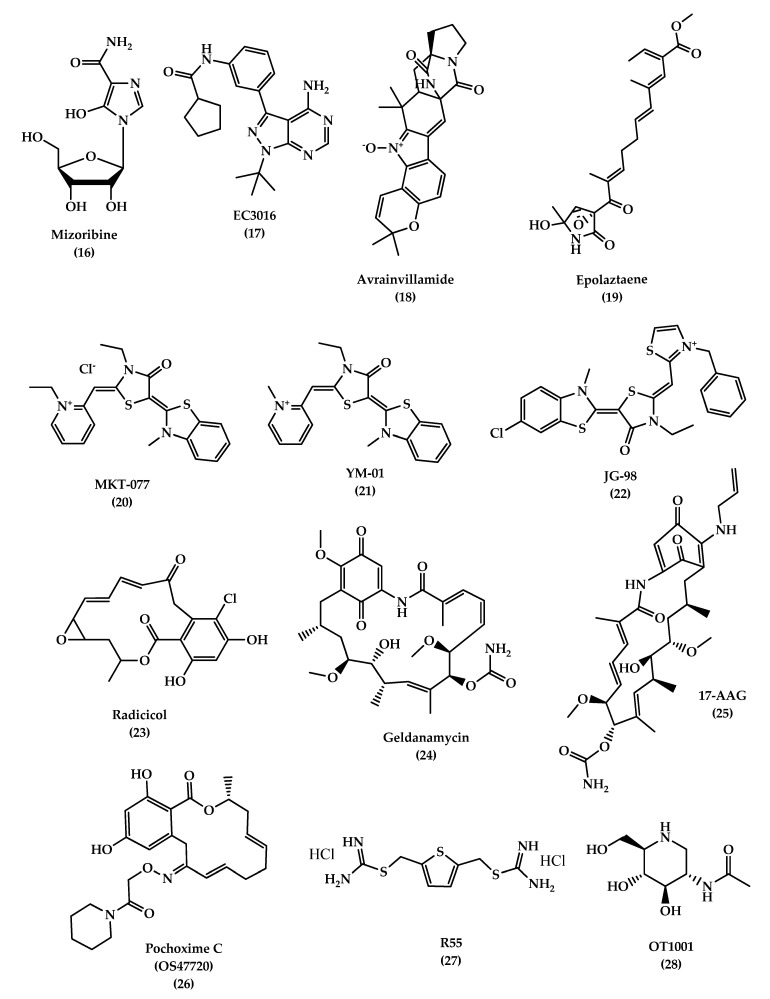
The chemical structures of different chaperone molecules: Mizoribine **16**, EC3016 **17**, Avrainvillamide **18**, Epolaztaene **19**, MKT-077 **20**, YM-01 **21**, JG-98 **22**, Radicicol **23**, Geldanamycin **24**, 17-AAG **25**, Pochoxime C (OS47720) **26**, R55 **27**, and OT1001 **28**.

Hsp60 plays an important role in mitochondrial protein folding. Its role in AD is not clear, some believe that the protein has a protective role and others think it has a harmful effect where it can be over-expressed by activated microglia, which increases pro-inflammatory factors such as toll-like receptor 4 (TLR-4) that stimulate neuronal cell death. Therefore, inhibiting activated microglia and Hsp60 expression is a promising strategy for preventing neurodegenerative diseases. Examples of compounds that inhibit Hsp60 are mizoribine (Immunosuppressant) (**16**, Figure 5) and pyrazolopyrimidine EC3016 (**17**, Figure 5). Both compounds act by blocking ATPase activity of Hsp60 and inhibiting protein folding. On the other hand, avrainvillamide, a fungal metabolite (**18**, Figure 5), and epolactaene, a bacterial metabolite (**19**, Figure 5), act by binding to the Hsp60′s cysteine residues and inhibit its folding activity. However, Hsp60’s role in AD remains controversial and there is a need for more investigations to understand its role [130].

2. Hsp70

Studies have shown that Hsp70 binds to Aβ42 and prevents self-aggregation. Martín-Peña et al. studied two isoforms of Hsp70, cytosolic and extracellular, in *Drosophila* flies AD models and evaluated their protective role against memory decline that results from Aβ42 aggregation. The animal studies showed that Hsp70 has a dual function: intracellularly and extracellularly, where it protects against Aβ42 neurotoxicity and synaptic loss. In addition to its ability to bind to tau and its hyper-phosphorylated form and prevent its formation, it decreases aggregation and promotes tau binding to microtubules. Hsp70 acts by activating microglia, insulin-degrading enzyme, and tumor growth facto*r-*β1, which degrades β-amyloids and prevents memory impairments [132,133]. Some studies in AD brain tissue demonstrated an overexpression of Hsp70 levels and a correlation with the presence of activated glia and stressed neurons. Also, it was found that Hsp70 is associated with extracellular deposits in AD. Drug therapies targeting Hsp70, mainly referring to previous anticancer drugs which target and inhibit Hsp70 ATP-binding site, are considered as candidates in AD treatment due to their ability to reduce tau levels in vitro and ex vivo. MKT-077(1-ethyl-2-((*Z*)-((*E*)-3-ethyl-5-(3-methylbenzo [*d*]thiazol-2(3*H*)-ylidene)-4-oxothiazolidin-2-ylidene)methyl)pyridin-1-ium chloride) (**20**, Figure 5), is an anticancer rhodacyanine compound that binds to mortalin, a mitochondrial Hsp70 site, and acts as an anti-proliferative agent, but the use of this compound was stopped due to toxicity side effects and low BBB penetration. On the other hand, YM-01 (**21**, Figure 5), a more potent MKT-077 derivative, was developed with a single replacement of the ethyl group on the pyridinium nitrogen of MKT-077 with a methyl group. JG-98 (**22**, Figure 5) is also an MKT-077 derivative with a 60-fold higher binding affinity to Hsp70 than YM-01 [130,134,135,136].

3. Hsp90

Hsp90 is another type of HSP that regulates the tau phosphorylation and dephosphorylation. An inhibition of Hsp90 results in a decrease in phosphorylation of tau due to a reduction in tau kinases, which is thought to be responsible for tau pathogenesis when it is hyperactivated. Hsp90 inhibitors are used for cancer therapy, but recently, they are considered as promising therapy for AD. Radicicol (RDC) (**23**, Figure 5) and geldanamycin (GA) (**24**, Figure 5) are Hsp90 inhibitors. GA is a natural antifungal compound and the first discovered Hsp90 inhibitor. Studies on this inhibitor were stopped due to its toxicity. On the other hand, 17-AAG (17-(Allylamino)-17-demethoxygeldanamycin) (**25**, Figure 5) is a GA derivative with a lower toxicity and better pharmacokinetic profile that showed a good improvement of the cognitive function by inducing other HSPs, like Hsp70, in addition to reducing NFTs in the transgenic mouse model by blocking the tau phosphorylation pathway, indirectly [137,138]. Pochoxime C (OS47720) (**26**, Figure 5) is also a CNS-permeable Hsp90 inhibitor that showed good safety and efficacy profiles when tested in the AD mouse model. Studies revealed that OS47720 acts by strengthening synaptic function via heat shock factor (HSF-1) activation and dependent transcriptional events [139].

The combined studies demonstrate that targeting HSPs is a promising strategy to develop drugs with a new mechanism of action for reducing pathogenic tau levels and restoring normal tau homeostasis.

- Vacuolar sorting protein 35 (VPS35)

An accumulation of proteins in neurons and glial cells leads to disturbance of cellular protein homeostasis. The endosomal-lysosomal system is responsible for transporting proteins for recycling and degradation. Any malfunction in the system can lead to several diseases, such as Alzheimer’s disease. Retromer is a complex of regulator proteins composed of sorting nexin (SNX1, 2, 5, 6) and vacuolar sorting proteins (VPS 26, 29, 35), which are responsible for transporting cargo molecules from the endosome to the *trans*-Golgi network. A loss of retromer’s function results in the downregulation of VPS35, which can increase Aβ formation, induce cognitive impairments, and cause synaptic dysfunction, which is reported in AD patients [140,141]. A study on 3xTg mice brains was conducted to evaluate the effect of VPS35 overexpression on memory function. The study showed that a significant reduction of the Aβ peptide and tau neuropathology (soluble, insoluble, and phosphorylated tau) was associated with overexpression of VPS35, in addition to a reduction in neuroinflammation and ameliorating synaptic dysfunction [142]. Therefore, VPS35 is an important promising therapeutic target for AD treatment. A small pharmacological chaperones molecule called R55 (thiophene-2,5-diylbis(methylene) dicarbamimidothioatedihydrochloride) (**27**, Figure 5), a thiophenethiourea derivative, can enhance retromer stability and function by increasing retromer proteins, shifting AOO from the endosome, and reducing pathogenic processing of APP, which may serve as a promising therapeutic molecule for neurodegenerative diseases [143].

- OT1001

Studies demonstrated that the accumulation of gangliosides has been associated with misfolding and aggregation of proteins in neurodegenerative diseases. Abnormal levels of mono-sialoganglioside (GM1, GM2, and GM3) have been reported in AD brains. Mutant forms of Aβ, like Dutch mutant APPE693Q, showed susceptibility to pro-aggregation properties of GM2 and GM3, resulting in the formation of Aβ peptides complexes with gangliosides (ganglioside-bound Aβ (GAβ) peptide) and subsequently leading to an acceleration of aggregation and accumulation of Aβ peptides.

β-hexosaminidase (β-hex) is a lysosomal enzyme that acts by catabolizing GM2 ganglioside, and increasing its activity can lead to a reduction of GM2 levels and Aβ aggregation and accumulation. Small molecules like pharmacological chaperones (PC) can selectively bind and stabilize wild-type proteins and restore their normal folding. OT1001 (**28**, Figure 5) is an iminosugar PC that targets β-hex and increases its level in the brain and reduces GAβ pathology. Studies on Dutch APPE693Q transgenic mice showed that OT1001 has good pharmacokinetics, brain penetration ability, and tolerability, with lower side effects. These make the compound a good drug candidate for increasing the β-hex activity [144].

#### 6.2.3. Natural Extract

For a long time, natural compounds have been used as therapeutic agents for several pathological diseases, and recent studies showed that they possess a neuroprotective effect. In vitro and in vivo studies have proven that natural compounds possess a therapeutic potential for AD, which allowed some of them to enter the clinical trials stages. Nicotine was the first natural compound entered in the clinical trials for AD, then other compounds like vitamins C, E, and D gained more attention and interest due to their protective role against neuroinflammation and oxidative damage. Recently, bryostatin, a macrolide lactone extract from *bryozoan Bugula neritina,* has been evaluated and showed the ability to induce α-secretase activity, reduce Aβ production, and enhance the learning and memory in an AD mice model [145]. Other natural compounds used in folk medicine (traditional Chinese medicine (TCM)) demonstrated a great potential in treating AD by acting on several mechanisms, as shown in Table 2 below [146].

## 7. Conclusions

Alzheimer’s disease is now considered a world health concern; as a consequence, the National Institute on Aging—Alzheimer’s Association reclassified and updated the 1984 NINCDS-ADRDA criteria for higher specificity, sensitivity, and early identification of patients at risk of developing AD. Several criteria have been proposed for a more accurate diagnosis of AD, including clinical biomarkers, bodily fluids, and imaging studies. Despite that, the treatment of AD remains symptomatic, without alteration in the disease’s prognosis. Inhibitors to cholinesterase enzyme such as galantamine, donepezil, and rivastigmine, and NMDA antagonists such as memantine, improve memory and alertness but do not prevent progression. Several studies have shown that modification in lifestyle habits like diet and exercise can improve brain health and reduce AD without medical intervention and is considered as a first-line intervention for all AD patients. Recently, the research is focusing on targeting the pathological features of AD such as Aβ and p-tau. Future therapies such as disease-modifying treatment can alter the progression of AD by targeting the Aβ pathway, and many drugs have entered the clinical trials, like AN-1792, solanezumab, bapineuzumab, semagacestat, avagacestat, and tarenflurbil, but failed in demonstrating efficacy in the final clinical stages. Other DMTs are still under investigation, such as those targeting Aβ and tau pathologies, such as aducanumab, gantenerumab, crenezumab, tideglusib, lithium, and others. Other promising compounds called chaperones like heat shock proteins and vacuolar sorting protein 35 (VPS35) function by assisting other proteins to function normally and to arrive at their destination in the cell safely, and therefore can be used as a treatment for neurodegenerative diseases. Moreover, the natural extracts used in folk Chinese medicine showed great potential in treating AD by acting on several mechanisms’ pathways. In conclusion, the success of AD treatment depends on its early administration and patient monitoring for disease progression using biomarkers diagnosis. Future therapies that target tau pathology and the use of combination therapy may have a potential to slow the progression of AD pathology. Designing a potent, selective, and effective drug is urgently needed to treat patients with AD and those at risk for developing the disease.

## Figures and Tables

**Table 1:** Disease modifying agents for the treatment of Alzheimer’s disease in clinical trials.

Disease Modifying Agents	Mechanism of Action
Phase 3 Clinical Trials	
**Aducanumab**	Monoclonal antibody—targets β-amyloid and removes it.
**Gantenerumab**	Monoclonal antibody—binds and removes β-amyloid.
**CAD106b**	Amyloid vaccine—stimulates production of antibodies against β-amyloid.
**BAN2401**	Monoclonal antibody—reduces protofibrillar β-amyloid.
**TRx0237 (LMTX)**	Tau protein aggregation inhibitor.
**AGB101**	Low-dose levetiracetam—improves synaptic function and reduces amyloid-induced neuronal hyperactivity
**ALZT-OP1 (cromolyn + ibuprofen)**	Mast cell stabilizer and anti-inflammatory—promotes microglial clearance of amyloid
**Azeliragon**	RAGE (Receptor for Advanced Glycation End-products) antagonist—reduces inflammation and amyloid transport into the brain
**BHV4157 (troriluzole)**	Glutamate modulator—reduces synaptic levels of glutamate and improves synaptic functioning
**Masitinib**	Tyrosine kinase inhibitor—modulates inflammatory mast cell and reduces amyloid protein and tau phosphorylation
**Phase 2 Clinical Trials**	
**Crenezumab**	Monoclonal antibody—targets soluble oligomers and removes β-amyloid
**ABBV-8E12**	Monoclonal antibody—prevents tau propagation
**ABvac40**	Active immunotherapy—targets β-amyloid and removes it
**BAN2401**	Monoclonal antibody—removes amyloid protofibrils and reduces amyloid plaques
**BIIB092**	Monoclonal antibody—removes tau and reduces tau propagation
**LY3002813 (donanemab)**	Monoclonal antibody—removes amyloid by recognizing aggregated pyroglutamate form of Aβ
**LY3303560 (zagotenemab)**	Monoclonal antibody—neutralizes soluble tau aggregates
**Semorinemab (RO7105705)**	Monoclonal antibody—removes extracellular tau
**APH-1105**	Alpha-secretase modulator—reduces amyloid
**Daratumumab**	Monoclonal antibody—immunomodulatory that targets CD38 and regulates microglial activity
**Dasatinib + Quercetin**	Tyrosine kinase inhibitor (dasatinib) + flavonoid (quercetin)—reduces senescent cells and tau aggregation
**IONIS MAPTRx (BIIB080)**	Epigenetic, Tau Antisense oligonucleotide—reduces tau production
**Lithium**	Neurotransmitter receptors ion channel modulator—improves neuropsychiatric symptoms
**Nilotinib**	Tyrosine kinase inhibitor—promotes clearance of amyloid and tau proteins
**Posiphen**	Selective inhibitor of APP—reduces amyloid, tau, and α-synuclein production
**PTI-125**	Filamin A protein inhibitor—reduces tau hyperphosphorylation, synaptic dysfunction, and stabilizes soluble amyloid and the α7 nicotinic acetylcholine receptor interaction
**PQ912**	Glutaminyl cyclase (QC) enzyme inhibitor—reduces amyloid plaques and pyroglutamates Aβ production
**Riluzole**	Glutamate receptor antagonist—reduces glutamate-mediated excitotoxicity
**Thiethylperazine (TEP)**	Activates ABCC1 (ATP binding cassette subfamily C member 1 transport protein)—removes amyloid
**Phase 1 Clinical Trials**	
**BIIB076**	Monoclonal antibody—removes tau and reduces tau propagation
**Lu AF87908**	Monoclonal antibody—removes tau
**anle138b**	Aggregation inhibitor—reduces tau aggregation
**RO7126209**	Monoclonal antibody—removes amyloid
**TPI-287**	Stabilizes tubulin-binding, microtubule, and reduces cellular damage mediated by tau

**Table 2:** Natural compounds used in folk medicine and their mechanism of actions.

Natural Compounds	Mechanism of Action
**Schisantherin A, Ginsenoside Rh2, and Angelica sinensis extracts**	Aβ formation inhibitors
**Shengmai (SM) formula, Uncarinic acid C, and** **Tanshinone IIA (TIIA) extract**	Reduction of Aβ accumulation
**Onjisaponin B, Notoginsenoside R1, and delta-9-Tetrahydrocannabinol (THC)/cannabidiol (CBD)**	Promotion of Aβ degradation
**Rhynchophylline (RIN), INM-176 (ethanolic extract of *Angelica gigas*)*, Houttuyniacordata* Thunb. (Saururaceae) water extracts, Huperzine A, and ethyl acetate extract from *Diospyros kaki* L.f**	Inhibition of Aβ Neurotoxicity and reduce over-activation of microglial cells, neuroinflammation, oxidative stress, and disruption of calcium homeostasis, which lead to neuron loss
**Tongmai Yizhi Decoction (TYD) (which includes six raw materials: safflower yellow (SY) from *Carthamustinctorius* L., geniposide from the fruit of *G. jasminoides* J. Ellis, ginsenoside Rd from *Panax ginseng* C. A. Mey, crocin from *Crocus sativus* L., and quinones)**	Inhibition of hyperphosphorylated tau protein and its aggregation

## References

[1] De-Paula V.J., Radanovic M., Diniz B.S., Forlenza O.V. (2012). Alzheimer’s disease. Sub-Cell. Biochem..

[2] Cipriani G., Dolciotti C., Picchi L., Bonuccelli U. (2011). Alzheimer and his disease: A brief history. Neurol. Sci. Off. J. Ital. Neurol. Soc. Ital. Soc. Clin. Neurophysiol..

[3] Blass J.P. (1985). Alzheimer’s disease. Dis. A Mon. Dm.

[4] Terry R.D., Davies P. (1980). Dementia of the Alzheimer type. Annu. Rev. Neurosci..

[5] Rathmann K.L., Conner C.S. (1984). Alzheimer’s disease: Clinical features, pathogenesis, and treatment. Drug Intell. Clin. Pharm..

[6] Yiannopoulou K.G., Papageorgiou S.G. (2020). Current and future treatments in alzheimer disease: An update. J. Cent. Nerv. Syst. Dis..

[7] Livingston G., Huntley J., Sommerlad A., Ames D., Ballard C., Banerjee S., Brayne C., Burns A., Cohen-Mansfield J., Cooper C. (2020). Dementia prevention, intervention, and care: 2020 report of the Lancet Commission. Lancet.

[8] Schachter A.S., Davis K.L. (2000). Alzheimer’s disease. Dialogues Clin. Neurosci..

[9] Jatoi S., Hafeez A., Riaz S.U., Ali A., Ghauri M.I., Zehra M. (2020). Low Vitamin B12 levels: An underestimated cause of minimal cognitive impairment and dementia. Cureus.

[10] Cho H.S., Huang L.K., Lee Y.T., Chan L., Hong C.T. (2018). Suboptimal baseline serum Vitamin B12 is associated with cognitive decline in people with Alzheimer’s disease undergoing cholinesterase inhibitor treatment. Front. Neurol..

[11] McKhann G., Drachman D., Folstein M., Katzman R., Price D., Stadlan E.M. (1984). Clinical diagnosis of Alzheimer’s disease: Report of the NINCDS-ADRDA Work Group under the auspices of Department of Health and Human Services Task Force on Alzheimer’s Disease. Neurology.

[12] Neugroschl J., Wang S. (2011). Alzheimer’s disease: Diagnosis and treatment across the spectrum of disease severity. Mt. Sinai J. Med. N. Y..

[13] McKhann G.M., Knopman D.S., Chertkow H., Hyman B.T., Jack C.R., Kawas C.H., Klunk W.E., Koroshetz W.J., Manly J.J., Mayeux R. (2011). The diagnosis of dementia due to Alzheimer’s disease: Recommendations from the National Institute on Aging-Alzheimer’s Association workgroups on diagnostic guidelines for Alzheimer’s disease. Alzheimer’s Dement. J. Alzheimer’s Assoc..

[14] Mayeux R., Stern Y. (2012). Epidemiology of Alzheimer disease. Cold Spring Harb. Perspect. Med..

[15] Yaari R., Fleisher A.S., Tariot P.N. (2011). Updates to diagnostic guidelines for Alzheimer’s disease. Prim. Care Companion Cns Disord..

[16] Serrano-Pozo A., Frosch M.P., Masliah E., Hyman B.T. (2011). Neuropathological alterations in Alzheimer disease. Cold Spring Harb. Perspect. Med..

[17] Spires-Jones T.L., Hyman B.T. (2014). The intersection of amyloid beta and tau at synapses in Alzheimer’s disease. Neuron.

[18] Singh S.K., Srivastav S., Yadav A.K., Srikrishna S., Perry G. (2016). Overview of Alzheimer’s disease and some therapeutic approaches targeting abeta by using several synthetic and herbal compounds. Oxidative Med. Cell. Longev..

[19] Cras P., Kawai M., Lowery D., Gonzalez-DeWhitt P., Greenberg B., Perry G. (1991). Senile plaque neurites in Alzheimer disease accumulate amyloid precursor protein. Proc. Natl. Acad. Sci. USA.

[20] Perl D.P. (2010). Neuropathology of Alzheimer’s disease. Mt. Sinai J. Med. N. Y..

[21] Armstrong R.A. (2009). The molecular biology of senile plaques and neurofibrillary tangles in Alzheimer’s disease. Folia Neuropathol..

[22] Chen G.F., Xu T.H., Yan Y., Zhou Y.R., Jiang Y., Melcher K., Xu H.E. (2017). Amyloid beta: Structure, biology and structure-based therapeutic development. Acta Pharmacol. Sin..

[23] Tabaton M., Piccini A. (2005). Role of water-soluble amyloid-beta in the pathogenesis of Alzheimer’s disease. Int. J. Exp. Pathol..

[24] Brion J.P. (1998). Neurofibrillary tangles and Alzheimer’s disease. Eur. Neurol..

[25] Metaxas A., Kempf S.J. (2016). Neurofibrillary tangles in Alzheimer’s disease: Elucidation of the molecular mechanism by immunohistochemistry and tau protein phospho-proteomics. Neural Regen. Res..

[26] Overk C.R., Masliah E. (2014). Pathogenesis of synaptic degeneration in Alzheimer’s disease and Lewy body disease. Biochem Pharm..

[27] Lleo A., Nunez-Llaves R., Alcolea D., Chiva C., Balateu-Panos D., Colom-Cadena M., Gomez-Giro G., Munoz L., Querol-Vilaseca M., Pegueroles J. (2019). Changes in synaptic proteins precede neurodegeneration markers in preclinical Alzheimer’s disease cerebrospinal fluid. Mol. Cell. Proteom. Mcp.

[28] Tarawneh R., D’Angelo G., Crimmins D., Herries E., Griest T., Fagan A.M., Zipfel G.J., Ladenson J.H., Morris J.C., Holtzman D.M. (2016). Diagnostic and prognostic utility of the synaptic marker neurogranin in Alzheimer Disease. JAMA Neurol..

[29] Dubois B., Hampel H., Feldman H.H., Scheltens P., Aisen P., Andrieu S., Bakardjian H., Benali H., Bertram L., Blennow K. (2016). Preclinical Alzheimer’s disease: Definition, natural history, and diagnostic criteria. Alzheimer’s Dement. J. Alzheimer’s Assoc..

[30] Kumar A., Sidhu J., Goyal A. (2020). Alzheimer Disease. StatPearls.

[31] Wattmo C., Minthon L., Wallin A.K. (2016). Mild versus moderate stages of Alzheimer’s disease: Three-year outcomes in a routine clinical setting of cholinesterase inhibitor therapy. Alzheimer’s Res. Ther..

[32] Apostolova L.G. (2016). Alzheimer disease. Continuum.

[33] Armstrong R.A. (2019). Risk factors for Alzheimer’s disease. Folia Neuropathol..

[34] Anand P., Singh B. (2013). A review on cholinesterase inhibitors for Alzheimer’s disease. Arch. Pharmacal Res..

[35] Babic T. (1999). The cholinergic hypothesis of Alzheimer’s disease: A review of progress. J. Neurol. Neurosurg. Psychiatry.

[36] Ferreira-Vieira T.H., Guimaraes I.M., Silva F.R., Ribeiro F.M. (2016). Alzheimer’s disease: Targeting the Cholinergic System. Curr. Neuropharmacol..

[37] Monczor M. (2005). Diagnosis and treatment of Alzheimer’s disease. Curr. Med. Chem. Cent. Nerv. Syst. Agents.

[38] Hampel H., Mesulam M.M., Cuello A.C., Farlow M.R., Giacobini E., Grossberg G.T., Khachaturian A.S., Vergallo A., Cavedo E., Snyder P.J. (2018). The cholinergic system in the pathophysiology and treatment of Alzheimer’s disease. Brain A J. Neurol..

[39] Paroni G., Bisceglia P., Seripa D. (2019). Understanding the amyloid hypothesis in Alzheimer’s disease. J. Alzheimer’s Dis. Jad.

[40] Kametani F., Hasegawa M. (2018). Reconsideration of amyloid hypothesis and tau hypothesis in Alzheimer’s disease. Front. Neurosci..

[41] Ricciarelli R., Fedele E. (2017). The amyloid cascade hypothesis in Alzheimer’s disease: It’s time to change our mind. Curr. Neuropharmacol..

[42] Guerreiro R., Bras J. (2015). The age factor in Alzheimer’s disease. Genome Med..

[43] Riedel B.C., Thompson P.M., Brinton R.D. (2016). Age, APOE and sex: Triad of risk of Alzheimer’s disease. J. Steroid Biochem. Mol. Biol..

[44] Hou Y., Dan X., Babbar M., Wei Y., Hasselbalch S.G., Croteau D.L., Bohr V.A. (2019). Ageing as a risk factor for neurodegenerative disease. Nat. Rev. Neurol..

[45] Bekris L.M., Yu C.E., Bird T.D., Tsuang D.W. (2010). Genetics of Alzheimer disease. J. Geriatr. Psychiatry Neurol..

[46] Van Cauwenberghe C., Van Broeckhoven C., Sleegers K. (2016). The genetic landscape of Alzheimer disease: Clinical implications and perspectives. Genet. Med. Off. J. Am. Coll. Med Genet..

[47] Khanahmadi M., Farhud D.D., Malmir M. (2015). Genetic of Alzheimer’s disease: A narrative review article. Iran. J. Public Health.

[48] Li N.M., Liu K.F., Qiu Y.J., Zhang H.H., Nakanishi H., Qing H. (2019). Mutations of beta-amyloid precursor protein alter the consequence of Alzheimer’s disease pathogenesis. Neural Regen. Res..

[49] Tcw J., Goate A.M. (2017). Genetics of beta-Amyloid precursor protein in Alzheimer’s disease. Cold Spring Harb. Perspect. Med..

[50] Bi C., Bi S., Li B. (2019). Processing of mutant beta-amyloid precursor protein and the clinicopathological features of familial Alzheimer’s disease. Aging Dis..

[51] Dai M.H., Zheng H., Zeng L.D., Zhang Y. (2018). The genes associated with early-onset Alzheimer’s disease. Oncotarget.

[52] Zhao J., Liu X., Xia W., Zhang Y., Wang C. (2020). Targeting amyloidogenic processing of APP in Alzheimer’s disease. Front. Mol. Neurosci..

[53] Cai Y., An S.S., Kim S. (2015). Mutations in presenilin 2 and its implications in Alzheimer’s disease and other dementia-associated disorders. Clin. Interv. Aging.

[54] Lanoiselee H.M., Nicolas G., Wallon D., Rovelet-Lecrux A., Lacour M., Rousseau S., Richard A.C., Pasquier F., Rollin-Sillaire A., Martinaud O. (2017). APP, PSEN1, and PSEN2 mutations in early-onset Alzheimer disease: A genetic screening study of familial and sporadic cases. PLoS Med..

[55] De Strooper B. (2007). Loss-of-function presenilin mutations in Alzheimer disease. Talking Point on the role of presenilin mutations in Alzheimer disease. Embo Rep..

[56] Kelleher R.J., Shen J. (2017). Presenilin-1 mutations and Alzheimer’s disease. Proc. Natl. Acad. Sci. USA.

[57] Walker E.S., Martinez M., Brunkan A.L., Goate A. (2005). Presenilin 2 familial Alzheimer’s disease mutations result in partial loss of function and dramatic changes in Abeta 42/40 ratios. J. Neurochem..

[58] Kim J., Basak J.M., Holtzman D.M. (2009). The role of apolipoprotein E in Alzheimer’s disease. Neuron.

[59] Liu C.C., Liu C.C., Kanekiyo T., Xu H., Bu G. (2013). Apolipoprotein E and Alzheimer disease: Risk, mechanisms and therapy. Nat. Rev. Neurol..

[60] Giau V.V., Bagyinszky E., An S.S., Kim S.Y. (2015). Role of apolipoprotein E in neurodegenerative diseases. Neuropsychiatr. Dis. Treat..

[61] Koldamova R., Fitz N.F., Lefterov I. (2014). ATP-binding cassette transporter A1: From metabolism to neurodegeneration. Neurobiol. Dis..

[62] Nordestgaard L.T., Tybjaerg-Hansen A., Nordestgaard B.G., Frikke-Schmidt R. (2015). Loss-of-function mutation in ABCA1 and risk of Alzheimer’s disease and cerebrovascular disease. Alzheimer’s Dement. J. Alzheimer’s Assoc..

[63] Foster E.M., Dangla-Valls A., Lovestone S., Ribe E.M., Buckley N.J. (2019). Clusterin in Alzheimer’s disease: Mechanisms, genetics, and lessons from other pathologies. Front. Neurosci..

[64] Holler C.J., Davis P.R., Beckett T.L., Platt T.L., Webb R.L., Head E., Murphy M.P. (2014). Bridging integrator 1 (BIN1) protein expression increases in the Alzheimer’s disease brain and correlates with neurofibrillary tangle pathology. J. Alzheimer’s Dis. Jad.

[65] Andrew R.J., De Rossi P., Nguyen P., Kowalski H.R., Recupero A.J., Guerbette T., Krause S.V., Rice R.C., Laury-Kleintop L., Wagner S.L. (2019). Reduction of the expression of the late-onset Alzheimer’s disease (AD) risk-factor BIN1 does not affect amyloid pathology in an AD mouse model. J. Biol. Chem..

[66] Soler-Lopez M., Badiola N., Zanzoni A., Aloy P. (2012). Towards Alzheimer’s root cause: ECSIT as an integrating hub between oxidative stress, inflammation and mitochondrial dysfunction. Hypothetical role of the adapter protein ECSIT in familial and sporadic Alzheimer’s disease pathogenesis. Bioessays News Rev. Mol. Cell. Dev. Biol..

[67] Mi Wi S., Park J., Shim J.H., Chun E., Lee K.Y. (2015). Ubiquitination of ECSIT is crucial for the activation of p65/p50 NF-kappaBs in Toll-like receptor 4 signaling. Mol. Biol. Cell.

[68] Soler-Lopez M., Zanzoni A., Lluis R., Stelzl U., Aloy P. (2011). Interactome mapping suggests new mechanistic details underlying Alzheimer’s disease. Genome Res..

[69] Zhao L., Woody S.K., Chhibber A. (2015). Estrogen receptor beta in Alzheimer’s disease: From mechanisms to therapeutics. Ageing Res. Rev..

[70] Sundermann E.E., Maki P.M., Bishop J.R. (2010). A review of estrogen receptor alpha gene (ESR1) polymorphisms, mood, and cognition. Menopause.

[71] Yaffe K., Lindquist K., Sen S., Cauley J., Ferrell R., Penninx B., Harris T., Li R., Cummings S.R. (2009). Estrogen receptor genotype and risk of cognitive impairment in elders: Findings from the Health ABC study. Neurobiol. Aging.

[72] Goumidi L., Dahlman-Wright K., Tapia-Paez I., Matsson H., Pasquier F., Amouyel P., Kere J., Lambert J.C., Meirhaeghe A. (2011). Study of estrogen receptor-alpha and receptor-beta gene polymorphisms on Alzheimer’s disease. J. Alzheimer’s Dis. Jad.

[73] Khorram Khorshid H.R., Gozalpour E., Saliminejad K., Karimloo M., Ohadi M., Kamali K. (2013). Vitamin D Receptor (VDR) polymorphisms and late-onset Alzheimer’s disease: An association study. Iran. J. Public Health.

[74] Liu X., Jiao B., Shen L. (2018). The epigenetics of Alzheimer’s Disease: Factors and therapeutic implications. Front. Genet..

[75] Wainaina M.N., Chen Z., Zhong C. (2014). Environmental factors in the development and progression of late-onset Alzheimer’s disease. Neurosci. Bull..

[76] Grant W.B., Campbell A., Itzhaki R.F., Savory J. (2002). The significance of environmental factors in the etiology of Alzheimer’s disease. J. Alzheimer’s Dis. Jad.

[77] Moulton P.V., Yang W. (2012). Air pollution, oxidative stress, and Alzheimer’s disease. J. Environ. Public Health.

[78] Croze M.L., Zimmer L. (2018). Ozone atmospheric pollution and Alzheimer’s disease: From epidemiological facts to molecular mechanisms. J. Alzheimer’s Dis. Jad.

[79] Hu N., Yu J.T., Tan L., Wang Y.L., Sun L., Tan L. (2013). Nutrition and the risk of Alzheimer’s disease. Biomed. Res. Int..

[80] Abate G., Marziano M., Rungratanawanich W., Memo M., Uberti D. (2017). Nutrition and AGE-ing: Focusing on Alzheimer’s disease. Oxidative Med. Cell. Longev..

[81] Koyama A., Hashimoto M., Tanaka H., Fujise N., Matsushita M., Miyagawa Y., Hatada Y., Fukuhara R., Hasegawa N., Todani S. (2016). Malnutrition in Alzheimer’s disease, dementia with lewy bodies, and frontotemporal lobar degeneration: Comparison using serum albumin, total protein, and hemoglobin level. PLoS ONE.

[82] Adlard P.A., Bush A.I. (2006). Metals and Alzheimer’s disease. J. Alzheimer’s Dis. Jad.

[83] Colomina M.T., Peris-Sampedro F. (2017). Aluminum and Alzheimer’s disease. Adv. Neurobiol..

[84] Huat T.J., Camats-Perna J., Newcombe E.A., Valmas N., Kitazawa M., Medeiros R. (2019). Metal toxicity links to Alzheimer’s disease and neuroinflammation. J. Mol. Biol..

[85] Sochocka M., Zwolinska K., Leszek J. (2017). The infectious etiology of Alzheimer’s disease. Curr. Neuropharmacol..

[86] Fulop T., Itzhaki R.F., Balin B.J., Miklossy J., Barron A.E. (2018). Role of microbes in the development of Alzheimer’s disease: State of the art—An international symposium presented at the 2017 IAGG congress in San Francisco. Front. Genet..

[87] Muzambi R., Bhaskaran K., Brayne C., Smeeth L., Warren-Gash C. (2019). Common bacterial infections and risk of incident cognitive decline or dementia: A systematic review protocol. BMJ Open.

[88] Stampfer M.J. (2006). Cardiovascular disease and Alzheimer’s disease: Common links. J. Intern. Med..

[89] Santos C.Y., Snyder P.J., Wu W.C., Zhang M., Echeverria A., Alber J. (2017). Pathophysiologic relationship between Alzheimer’s disease, cerebrovascular disease, and cardiovascular risk: A review and synthesis. Alzheimer’s Dement..

[90] De Bruijn R.F., Ikram M.A. (2014). Cardiovascular risk factors and future risk of Alzheimer’s disease. BMC Med..

[91] Alford S., Patel D., Perakakis N., Mantzoros C.S. (2018). Obesity as a risk factor for Alzheimer’s disease: Weighing the evidence. Obes. Rev. Off. J. Int. Assoc. Study Obes..

[92] Pegueroles J., Jimenez A., Vilaplana E., Montal V., Carmona-Iragui M., Pane A., Alcolea D., Videla L., Casajoana A., Clarimon J. (2018). Obesity and Alzheimer’s disease, does the obesity paradox really exist? A magnetic resonance imaging study. Oncotarget.

[93] Anjum I., Fayyaz M., Wajid A., Sohail W., Ali A. (2018). Does obesity increase the risk of dementia: A literature review. Cureus.

[94] Lee H.J., Seo H.I., Cha H.Y., Yang Y.J., Kwon S.H., Yang S.J. (2018). Diabetes and Alzheimer’s disease: Mechanisms and nutritional aspects. Clin. Nutr. Res..

[95] Singh R., Sadiq N.M. (2020). Cholinesterase Inhibitors. StatPearls.

[96] Eldufani J., Blaise G. (2019). The role of acetylcholinesterase inhibitors such as neostigmine and rivastigmine on chronic pain and cognitive function in aging: A review of recent clinical applications. Alzheimers Dement.

[97] Sharma K. (2019). Cholinesterase inhibitors as Alzheimer’s therapeutics (Review). Mol. Med. Rep..

[98] Wang R., Reddy P.H. (2017). Role of glutamate and NMDA receptors in Alzheimer’s disease. J. Alzheimer’s Dis. Jad.

[99] Kuns B., Rosani A., Varghese D. (2020). Memantine. StatPearls.

[100] Briggs R., Kennelly S.P., O’Neill D. (2016). Drug treatments in Alzheimer’s disease. Clin. Med..

[101] Crous-Bou M., Minguillon C., Gramunt N., Molinuevo J.L. (2017). Alzheimer’s disease prevention: From risk factors to early intervention. Alzheimer’s Res. Ther..

[102] Crismon M.L. (1994). Tacrine: First drug approved for Alzheimer’s disease. Ann. Pharmacother..

[103] Qizilbash N., Birks J., Lopez Arrieta J., Lewington S., Szeto S. (2000). Tacrine for Alzheimer’s disease. Cochrane Database Syst. Rev..

[104] Cacabelos R. (2007). Donepezil in Alzheimer’s disease: From conventional trials to pharmacogenetics. Neuropsychiatr. Dis. Treat..

[105] Kumar A., Sharma S. (2020). Donepezil. StatPearls.

[106] Dooley M., Lamb H.M. (2000). Donepezil: A review of its use in Alzheimer’s disease. Drugs Aging.

[107] Annicchiarico R., Federici A., Pettenati C., Caltagirone C. (2007). Rivastigmine in Alzheimer’s disease: Cognitive function and quality of life. Ther. Clin. Risk Manag..

[108] Muller T. (2007). Rivastigmine in the treatment of patients with Alzheimer’s disease. Neuropsychiatr. Dis. Treat..

[109] Khoury R., Rajamanickam J., Grossberg G.T. (2018). An update on the safety of current therapies for Alzheimer’s disease: Focus on rivastigmine. Ther. Adv. Drug Saf..

[110] Birks J., Grimley Evans J., Iakovidou V., Tsolaki M., Holt F.E. (2009). Rivastigmine for Alzheimer’s disease. Cochrane Database Syst. Rev..

[111] Scott L.J., Goa K.L. (2000). Galantamine: A review of its use in Alzheimer’s disease. Drugs.

[112] Prvulovic D., Hampel H., Pantel J. (2010). Galantamine for Alzheimer’s disease. Expert Opin. Drug Metab. Toxicol..

[113] Kim J.K., Park S.U. (2017). Pharmacological aspects of galantamine for the treatment of Alzheimer’s disease. Excli J..

[114] Wahba S.M., Darwish A.S., Kamal S.M. (2016). Ceria-containing uncoated and coated hydroxyapatite-based galantamine nanocomposites for formidable treatment of Alzheimer’s disease in ovariectomized albino-rat model. Mater. Sci. Eng. C Mater. Biol. Appl..

[115] Liu J., Chang L., Song Y., Li H., Wu Y. (2019). The role of NMDA receptors in Alzheimer’s disease. Front. Neurosci..

[116] Huang Y.J., Lin C.H., Lane H.Y., Tsai G.E. (2012). NMDA Neurotransmission dysfunction in behavioral and psychological symptoms of Alzheimer’s disease. Curr. Neuropharmacol..

[117] Companys-Alemany J., Turcu A.L., Bellver-Sanchis A., Loza M.I., Brea J.M., Canudas A.M., Leiva R., Vazquez S., Pallas M., Grinan-Ferre C. (2020). A novel NMDA receptor antagonist protects against cognitive decline presented by senescent mice. Pharmaceutics.

[118] Folch J., Busquets O., Ettcheto M., Sanchez-Lopez E., Castro-Torres R.D., Verdaguer E., Garcia M.L., Olloquequi J., Casadesus G., Beas-Zarate C. (2018). Memantine for the treatment of dementia: A Review on its current and future applications. J. Alzheimer’s Dis. Jad.

[119] Cummings J., Fox N. (2017). Defining disease modifying therapy for Alzheimer’s Disease. J. Prev. Alzheimer’s Dis..

[120] Huang L.K., Chao S.P., Hu C.J. (2020). Clinical trials of new drugs for Alzheimer disease. J. Biomed. Sci..

[121] Neumann U., Ufer M., Jacobson L.H., Rouzade-Dominguez M.L., Huledal G., Kolly C., Luond R.M., Machauer R., Veenstra S.J., Hurth K. (2018). The BACE-1 inhibitor CNP520 for prevention trials in Alzheimer’s disease. Embo Mol. Med..

[122] Vandenberghe R., Riviere M.E., Caputo A., Sovago J., Maguire R.P., Farlow M., Marotta G., Sanchez-Valle R., Scheltens P., Ryan J.M. (2017). Active Abeta immunotherapy CAD106 in Alzheimer’s disease: A phase 2b study. Alzheimers Dement.

[123] Cummings J., Lee G., Ritter A., Sabbagh M., Zhong K. (2020). Alzheimer’s disease drug development pipeline: 2020. Alzheimers Dement.

[124] Tolar M., Abushakra S., Hey J.A., Porsteinsson A., Sabbagh M. (2020). Aducanumab, gantenerumab, BAN2401, and ALZ-801-the first wave of amyloid-targeting drugs for Alzheimer’s disease with potential for near term approval. Alzheimer’s Res. Ther..

[125] Galimberti D., Scarpini E. (2011). Disease-modifying treatments for Alzheimer’s disease. Ther. Adv. Neurol. Disord..

[126] Ghezzi L., Scarpini E., Galimberti D. (2013). Disease-modifying drugs in Alzheimer’s disease. Drug Des. Dev. Ther..

[127] Medina M. (2018). An Overview on the clinical development of tau-based therapeutics. Int. J. Mol. Sci..

[128] Davidowitz E.J., Krishnamurthy P.K., Lopez P., Jimenez H., Adrien L., Davies P., Moe J.G. (2020). In vivo validation of a small molecule inhibitor of tau self-association in htau mice. J. Alzheimer’s Dis. Jad.

[129] Cortez L., Sim V. (2014). The therapeutic potential of chemical chaperones in protein folding diseases. Prion.

[130] Campanella C., Pace A., Caruso Bavisotto C., Marzullo P., Marino Gammazza A., Buscemi S., Palumbo Piccionello A. (2018). Heat shock proteins in Alzheimer’s disease: Role and targeting. Int. J. Mol. Sci..

[131] Wilhelmus M.M., de Waal R.M., Verbeek M.M. (2007). Heat shock proteins and amateur chaperones in amyloid-Beta accumulation and clearance in Alzheimer’s disease. Mol. Neurobiol..

[132] Martin-Pena A., Rincon-Limas D.E., Fernandez-Funez P. (2018). Engineered Hsp70 chaperones prevent Abeta42-induced memory impairments in a Drosophila model of Alzheimer’s disease. Sci. Rep..

[133] Calderwood S.K., Murshid A. (2017). Molecular chaperone accumulation in cancer and decrease in Alzheimer’s disease: The potential roles of HSF1. Front. Neurosci..

[134] Repalli J., Meruelo D. (2015). Screening strategies to identify HSP70 modulators to treat Alzheimer’s disease. Drug Des. Dev. Ther..

[135] Li X., Shao H., Taylor I.R., Gestwicki J.E. (2016). Targeting allosteric control mechanisms in heat shock protein 70 (Hsp70). Curr. Top. Med. Chem..

[136] Abisambra J., Jinwal U.K., Miyata Y., Rogers J., Blair L., Li X., Seguin S.P., Wang L., Jin Y., Bacon J. (2013). Allosteric heat shock protein 70 inhibitors rapidly rescue synaptic plasticity deficits by reducing aberrant tau. Biol. Psychiatry.

[137] Bohush A., Bieganowski P., Filipek A. (2019). Hsp90 and its co-chaperones in neurodegenerative diseases. Int. J. Mol. Sci..

[138] Ou J.R., Tan M.S., Xie A.M., Yu J.T., Tan L. (2014). Heat shock protein 90 in Alzheimer’s disease. Biomed Res. Int..

[139] Wang B., Liu Y., Huang L., Chen J., Li J.J., Wang R., Kim E., Chen Y., Justicia C., Sakata K. (2017). A CNS-permeable Hsp90 inhibitor rescues synaptic dysfunction and memory loss in APP-overexpressing Alzheimer’s mouse model via an HSF1-mediated mechanism. Mol. Psychiatry.

[140] Li J.G., Chiu J., Ramanjulu M., Blass B.E., Pratico D. (2020). A pharmacological chaperone improves memory by reducing Abeta and tau neuropathology in a mouse model with plaques and tangles. Mol. Neurodegener..

[141] Vagnozzi A.N., Li J.G., Chiu J., Razmpour R., Warfield R., Ramirez S.H., Pratico D. (2019). VPS35 regulates tau phosphorylation and neuropathology in tauopathy. Mol. Psychiatry.

[142] Li J.G., Chiu J., Pratico D. (2020). Full recovery of the Alzheimer’s disease phenotype by gain of function of vacuolar protein sorting 35. Mol. Psychiatry.

[143] Mecozzi V.J., Berman D.E., Simoes S., Vetanovetz C., Awal M.R., Patel V.M., Schneider R.T., Petsko G.A., Ringe D., Small S.A. (2014). Pharmacological chaperones stabilize retromer to limit APP processing. Nat. Chem. Biol..

[144] Knight E.M., Williams H.N., Stevens A.C., Kim S.H., Kottwitz J.C., Morant A.D., Steele J.W., Klein W.L., Yanagisawa K., Boyd R.E. (2015). Evidence that small molecule enhancement of beta-hexosaminidase activity corrects the behavioral phenotype in Dutch APP(E693Q) mice through reduction of ganglioside-bound Abeta. Mol. Psychiatry.

[145] Andrade S., Ramalho M.J., Loureiro J.A., Pereira M.D.C. (2019). Natural compounds for Alzheimer’s disease therapy: A systematic review of preclinical and clinical studies. Int. J. Mol. Sci..

[146] Ma Y., Yang M.W., Li X.W., Yue J.W., Chen J.Z., Yang M.W., Huang X., Zhu L.L., Hong F.F., Yang S.L. (2019). Therapeutic effects of natural drugs on Alzheimer’s disease. Front. Pharmacol..

